# Real-world efficacy and safety of pyrotinib in patients with HER2-positive metastatic breast cancer: A prospective real-world study

**DOI:** 10.3389/fphar.2023.1100556

**Published:** 2023-03-21

**Authors:** Qiongwen Zhang, Ping He, Tinglun Tian, Xi Yan, Juan Huang, Zhang Zhang, Hong Zheng, Xiaorong Zhong, Ting Luo

**Affiliations:** ^1^ Department of Head and Neck Oncology, Department of Radiation Oncology, Cancer Center, and State Key Laboratory of Biotherapy, West China Hospital of Sichuan University, Chengdu, China; ^2^ Breast Disease Center, Cancer Center, West China Hospital, Sichuan University, Chengdu, China; ^3^ Department of Radiology, West China Hospital, Sichuan University, Chengdu, China; ^4^ Department of Pathology, West China Hospital, Sichuan University, Chengdu, China; ^5^ Multi-omics Laboratory of Breast Diseases, State Key Laboratory of Biotherapy, National Collaborative, Innovation Center for Biotherapy, West China Hospital, Sichuan University, Chengdu, China

**Keywords:** HER2-positive breast cancer, real-world studies, brain metastase, pyrotinib, tyrosine kinase inhibitors

## Abstract

**Background:** Pyrotinib, a novel irreversible EGFR/HER2 dual tyrosine kinase inhibitor, shows encouraging anticancer activity and acceptable tolerability in multiple phase II and phase III randomized clinical trials, but the real-world data of pyrotinib, especially the outcomes in HER2-positive metastatic breast cancer, have been rarely reported. Here, we evaluated the treatment outcomes of pyrotinib in real-world practice in patients with HER2-positive metastatic breast cancer (MBC).

**Methods:** This was a prospective, real-world, observational cohort study. Through the Breast Cancer Information Management System, HER-2 positive MBC patients treated with pyrotinib between 2017/06 and 2020/09 were included. Provider-reported objective response rate, progression-free survival (PFS), and overall survival (OS) were considered in the assessment of treatment outcomes. Tumor responses to pyrotinib treatment were calculated using RECIST 1.1. Adverse events were evaluated using clinical records.

**Results:** The trial involved 113 individuals who were receiving pyrotinib treatment, with an average age of 51 years. Complete response, partial response and stable disease were observed in 9 (8.0%), 66 (58.4%), and 17 (15.0%) patients, respectively, while progressive disease was recorded in 20 (17.7%) patients. After a median follow-up of 17.2 months, the median PFS was 14.1. The most common adverse events of any grade were diarrhea (87.6%), vomiting (31.9%), and palmar-plantar erythrodysesthesia (26.6%). Among the patients with brain metastases, the median PFS and OS were 15.2 and 19.8 months, respectively. In addition, pyrotinib has similar efficacy in various subtypes of HER2-positive MBC patients, as shown by the lack of a significant difference of PFS and OS among pyrotinib-treated patients with or without brain metastases, or patients using pyrotinib as first-line, second-line, third-line or beyond therapies.

**Conclusion:** Our real-world results demonstrated equivalent clinical efficacy in HER-2 positive MBC patients compared to phase II and phase III clinical trials with pyrotinib, and promising outcomes in patients with brain metastases.

## Introduction

Human epidermal growth factor receptor 2 (HER2) positive breast cancer accounts for approximately 20%–25% of all breast cancer and is associated with aggressive behavior and poor prognosis (Owens et al., 2004; Cronin et al., 2010). Despite available HER2-targeted drugs have dramatically improved outcomes in patients with HER2-positice breast cancer, resistance will eventually develop in the majority of patients (Bartsch and Bergen, 2018). Moreover, HER2-postive breast cancer are at high risk of developing metastatic disease, particularly in brain which may develop in up to half of patients (Gabos et al., 2006; Leyland-Jones, 2009; Olson et al., 2013; Martin et al., 2017). HER-2 targeted drugs can be broadly divided into three categories: the monoclonal antibodies trastuzumab and pertuzumab, the antibody-drug congates trastuzumab emansine (T-DM1) and trastuzumab deruxtecan (DS-8201), and small molecule tyrosine kinase inhibitors (TKIs) including lapatinib, neratinib, and tucatinib (Escrivá-de-Romaní et al., 2018; Collins et al., 2019; Conlon et al., 2021). The phase 3 randomized CLEOPATRA and EMILIA trials established trastuzumab plus pertuzumab and taxane as the first-line standard treatment for HER2-positive metastatic breast cancer, and T-DM1 as the second-and-beyond line treatment by the national comprehensive Cancer Network guideline (category 2A) (Giordano et al., 2018; Montemurro et al., 2020; Gradishar et al., 2021). Over the past few years, newer options for HER2+ MBC patients have emerged and approved by the United States (US) Food and Drug Administration (FDA) including trastuzumab deruxtecan (T-DXd) in December 2019 (in the US, fam-trastuzumab deruxtecan-nxki), neratinib in February 2020, and tucatinib in April 2020. However, during the study period, neratinib, tucatinib, and T-DM1 were not accessible to most Chinese patients with breast cancer.

Pyrotinib is an irreversible pan-ErbB receptor tyrosine kinase inhibitor (TKI) drug targeting epidermal growth factor receptor (EGFR), HER1, HER2, and HER4 (Zhu et al., 2016; Li et al., 2017), and it has shown promising antitumor activities in patients with HER2-positive metastatic breast cancer according to the results of phase I trials (Ma et al., 2017; Li et al., 2019). On the basis of these findings, pyrotinib in combination with capecitabine was firstly approved in China for metastatic breast cancer patients in August 2018 (Blair, 2018). The following phase II and phase III trials also have shown encouraging results for trastuzumab-resistant metastatic breast cancer patients (Ma et al., 2019; Yan et al., 2020; Xu et al., 2021). In PHENIX study, it revealed that pyrotinib plus capecitabine increased median progression-free survival (PFS) compared with placebo plus capecitabine (11.1 months vs. 4.1 months) in patients who had previously treated with trastuzumab and taxane for metastasis. (Yan et al., 2020). The PHOEBE study further showed that pyrotinib combined with capecitabine increased PFS by 5.7 months compared with lapatinib plus capecitabine (12.5 months vs. 6.8 months) for pathologically confirmed HER2-positive metastatic breast cancer patients, who had previously treated with trastuzumab and taxanes (Xu et al., 2021). In 2020, pyrotinib combined with capetabine was recommended as a second-line treatment (category 1A) by the Chinese Society of Clinical Oncology (CSCO) Breast Cancer Guideline for HER2-positive metastatic breast cancer. Although these encouraging results have confirmed the efficacy of pyrotinib for HER2-positive metastatic breast cancer, there is limited data on real-world clinical practice to assess the safety and effectiveness of pyrotinib.

In this study, we reported a clinical real-world outcome of HER2-positive metastasis breast cancer patients treated with pyrotinib-based therapy in a prospective cohort. We reported survival analysis and response rates in patients treated with pyrotinib. Then, the adverse events (AEs) associated with pyrotinib were also analyzed. Lastly, we further analyzed the efficacy of pyrotinib for patients with brain metastasis and patients using pyrotinib as first-line, second-line, third-line or beyond treatment.

## Materials and methods

### Data collection and study design

This study was designed as a China-based, single-center, prospective, real-world, observational cohort study. 382 HER-2 positive MBC patients diagnosed between 2017/06 and 2020/09 in West China Hospital were enrolled from the Breast Cancer Information Management System (BCIMS). The BCIMS, which began collecting real-world data from a breast cancer cohort in West China Hospital in 1989, has previously been reported (Peng et al., 2016; Hu et al., 2019). Eligibility criteria included 1) a histologic or cytologic diagnosis of MBC; 2) immunochemistry 3+, or immunochemistry 1/2 + together with HER2 gene amplification by fluorescence *in situ* hybridization; 3) a measurable lesion as defined by Response Evaluation Criteria in Solid Tumor 1.1 (RECIST 1.1); 4) a performance status of 0–1 on the Eastern Cooperative Oncology Group scale; 5) adequate bone marrow and organ functions; and 6) patients with complete and accurate media records. Patients were excluded if they did not have received pyrotinib treatment, or discontinued pyrotinib treatment, or lost treatment information, or lost to follow-up for other reasons. The data including detailed information on demographics, diagnosis, tumor characteristics, treatment information, and AEs was extracted from BCIMS, and were documented in an electronic case-report form.

The patients in the study were followed up according to the ESMO’s guidelines for metastasis breast cancer. Imaging follow-up was performed every two or four treatment cycles (21 days per cycle). The protocol, consent form, and study documents were approved by Biomedical Research Committee (approval number: 2012130), West China Hospital, Sichuan University and conducted in accordance with Helsinki Declaration of 1964, Good Clinical Practice guidelines, Chinese laws and regulatory requirements. Written informed consents were obtained from all included participants prior to enrollment.

### Anti-HER-2 therapy

All included patients received pyrotinib in 21-day cycles for metastasis. Patients in pyrotinib group were given continuous oral pyrotinib at a dose of 400 mg/d within 30 min after breakfast on days 1–14 of each cycle until disease progression, unmanageable toxic effects, death, withdrawal of consent, investigator decision, or study completion. Treatment delays and dose modifications were allowed to manage AEs. The dose reductions of pyrotinib were permitted stepwise from 400 to 320–240 mg, if pyrotinib-related AEs were experienced.

### Primary and secondary outcomes of interest

The primary end point was PFS, which was defined as the time form drug administration to disease progression, as assessed by investigator, according to RECIST 1.1 criteria. Secondary endpoints included objective response rate (ORR), overall survival (OS) and safety. A complete or partial response (CR or PR) required confirmation at least 4 weeks after the initial response. Treatment toxicity were assessed at each follow-up by using patients and clinical records according to the National Cancer Common Terminology Criteria for Adverse Events (NCI CTCAE v5.0).

### Statistical analysis

We first analyzed data for all included pyrotinib-treated patients. PFS and OS were estimated using Kaplan-Meier curves. Median survival time (median PFS and OS) and 95% confidence intervals (CIs) were calculated. Univariable and multivariate logistic regression models were used for assessment of the adjusted effects of variates on ORR. Meanwhile, the adjusted effects of variables on PFS were assessed using univariable and multivariable Cox proportional hazard models.

Next, to determine which subset of patients get more survival benefit from pyrotinib, we additionally performed the analysis of PFS and OS by dividing patients into subgroups: age at diagnosis (<40 or ≥40), brain metastasis (negative or positive), visceral metastasis (negative or positive) and previous treatment (with chemotherapy or without chemotherapy, with radiotherapy or without radiotherapy, with endocrinotherapy or without visceral therapy). All statistical analysis was performed in STATA (version 14.0; Stata Corporation).

## Results

### Patients’ clinical characteristics

**FIGURE 1:**
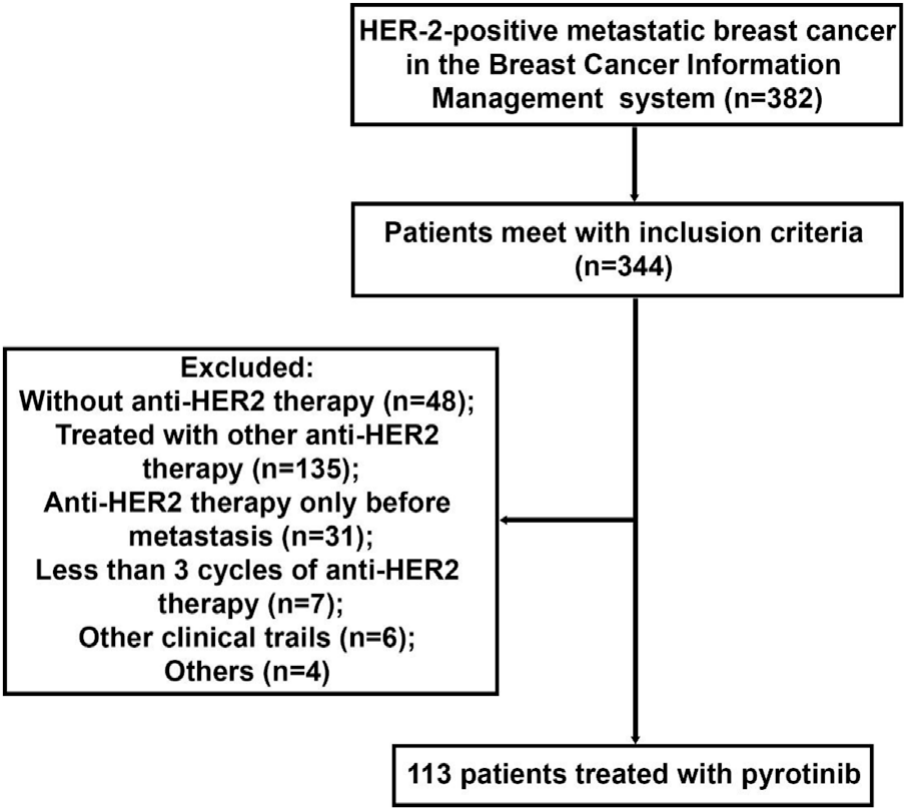
Flow diagram of HER-2 positive metastatic breast cancer included in the study.

The patients flow chart is shown in Figure 1. A total of 382 patients were initially enrolled from the BCIMS, and 344 patients met the inclusion criteria. Among these, 135 patients were treated with other anti-HER2 drugs except pyrotinib for metastasis. No anti-HER2 treatment were performed in 48 patients, and 31 patients received anti-HER2 treatment only before the metastasis. Seven patients had received less than 3 cycles of anti-HER2 therapy (3 with trastuzumab and 4 with pyrotinib), and 6 patients were involved in other clinical trials. There were another 4 patients excluded from this study: 2 patients lost to follow-up, and 2 withdraw drugs by themselves. The remaining 113 patients treated with pyrotinib were enrolled in subsequent analyses. The baseline characteristics of the 113 enrolled patients are shown in Table 1. Among the enrolled patients, 30 (26.55%) patients got brain metastasis, and 31 (27.43%) patients got more than 3 metastasis sites. Previous treatment included surgery in 89 (77.88%) of the patients, chemotherapy in 107 (94.69%), radiotherapy in 52 (46.02%) and endocrinotherapy in 44 (38.94%). 11 (9.73%) patients did not received anti-HER2 therapy before pyrotinib, while 102 (90.27%) patients got previous anti-HER2 therapy at different stage of the disease (24 only at early stage, 64 at advanced stage and 14 at both early and advanced stage). Twenty (17.70%), 61 (53.98%), and 32 (28.32%) of the enrolled patients received pyrotinib treatment for first line, second line, third line or beyond, respectively.

**TABLE 1:** The baseline characteristics of the 113 enrolled patients.

Characteristic	Patients (*N* = 113)
Median age at (range), years	51 (24–76)
<50	42 (37.17%)
≥50	71 (62.83%)
Menopause (%)	
No	61 (53.98%)
Yes	52 (46.02%)
Hormone Receptor Status (%)	
Unknown	—
Positive	65 (57.52%)
Negative	48 (42.48%)
HER2 Status (%)	
+++	88 (77.88%)
++ and FISH Amplification	25 (22.12%)
Metastasis site (%)	
Brain	30 (26.55%)
Lung	51 (45.13%)
Liver	44 (38.94%)
Bone only	9 (7.96%)
Other	15 (13.27%)
Number of metastasis (%)	
1	49 (43.36%)
2	33 (29.20%)
≥3	31 (27.43%)
Breast Cancer Related Surgery (%)	
No	25 (22.12%)
Yes	88 (77.88%)
Chemotherapy (%)	
No	6 (5.31%)
Yes	107 (94.69%)
Radiotherapy (%)	
No	61 (53.98%)
Yes	52 (46.02%)
Endocrinotherapy (%)	
No	69 (61.06%)
Yes	44 (38.94%)
Anti-HER2 Therapy (%)	
No	11 (9.73%)
Only early stage	23 (21.24%)
Only advanced stage	64 (56.64%)
Early and advanced stage	14 (12.39%)
Pyrotinib advanced anti-HER2 therapy lines (%)	
First Line	20 (17.70%)
Second Line	61 (53.98%)
Third Line and Beyond	32 (28.32%)

### Efficacy outcomes

**FIGURE 2:**
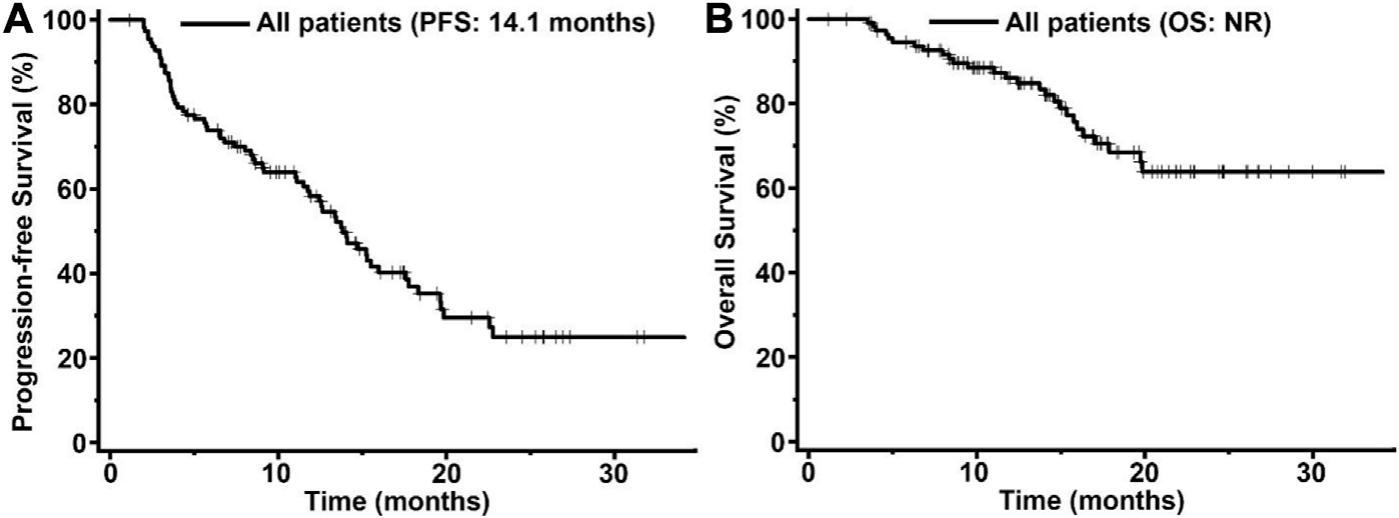
Kaplan-Meier curves of progression-free survival (PFS, **(A)** and overall survival (OS, **(B)** for patients with HER2-positive MBC treated with pyrotinib (*n* = 113). The tick marks indicate the time points at which the data were censored. NR, not reached.

In order to assessed the activity and safety of pyrotinib in HER2-positive MBC patients, we analyzed the PFS and OS in the 113 pyrotinib-treated patients (Figure 2). The median PFS was 14.1 months (95% confidence interval [CI] 12.5-17.8) with a median follow up of 17.2 months. However, the median OS was not reached in this study.

A total of 112 patients were included in ORR analysis, with one patient excluded because of lack of measurable lesions (Table 2). 9 (8.0%) patients achieved complete response (CR), and 66 (58.4%) patients achieved partial response (PR), resulting in an ORR of pyrotinib in this study at 66.4%.

**TABLE 2:** Response rate and disease progression in 113 patients treated with pyrotinib.

Best response	Patients (*N* = 113)
CR, no. (%)	9 (7.96%)
PR, no. (%)	66 (58.41%)
SD, no. (%)	17 (15.04%)
PD, no. (%)	20 (17.70%)
Missing, no. (%)	1 (0.88%)
Overall, no.	113
ORR,%(95%CI^a^)	66.37% (57.44%-75.56%)

^a^ 95%CI was obtained *via* exact Clopper-Pearson Method.

### Safety

As Table 3 displayed, the adverse events (AEs) of grade ≥3 were reported in 16 patients (15.5%). The most common AE in the pyrotinib-treated group was diarrhea (87.6%), but only 13 patients (11.5%) reported grade ≥3 diarrhea. Other common AEs of all grades that were documented in ≥15% pyrotinib-treated patients included vomiting (31.9%), palmar-plantar erythrodysesthesia (26.6%), nausea (18.6%), and mucositis oral (17.7%). No grade 4 or higher AEs were found and no treatment-related death was reported.

**TABLE 3:** Adverse events of matched-paired patients with different treatments.

Event	Pyrotinib (*N* = 113)			
	Any Grade	Grade 1	Grade 2	Grade 3
Diarrhea (%)	99 (87.61%)	49 (43.36%)	37 (32.74%)	13 (11.5%)
Vomiting (%)	36 (31.86%)	21 (18.58%)	15 (13.27%)	0
Palmar-plantar Erythrodysesthesia Syndrome (%)	30 (26.55%)	14 (12.39%)	14 (12.39%)	2 (1.77%)
Nausea (%)	21 (18.58%)	17 (15.04%)	4 (3.54%)	0
Mucositis Oral (%)	20 (17.70%)	14 (12.39%)	5 (4.42%)	1 (0.88%)
Rash (%)	16 (14.16%)	9 (7.96%)	7 (6.19%)	0
Malaise (%)	14 (12.39%)	14 (12.39%)	0	0
Abdominal Distension/Abdominal Pain (%)	12 (10.62%)	10 (8.85%)	2 (1.77%)	0
White Blood Cell Decreased (%)	6 (5.31%)	5 (4.42%)	1 (0.88%)	0
Anorexia (%)	8 (7.08%)	8 (7.08%)	0	0
Headache/Dizziness (%)	5 (4.42%)	5 (4.42%)	0	0
Constipation (%)	3 (2.65%)	3 (2.65%)	0	0
Liver Impairment (%)	2 (1.77%)	2 (1.77%)	0	0
Numbness of Teh Extremities (%)	3 (2.65%)	3 (2.65%)	0	0
Dryness of The Nasal Cavity (%)	2 (1.77%)	2 (1.77%)	0	0
Electrolyte Disorder (%)	2 (1.77%)	2 (1.77%)	0	0
Edema Limbs (%)	2 (1.77%)	2 (1.77%)	0	0
Menstruation (%)	2 (1.77%)	2 (1.77%)	0	0
Dry and Ulceration Mouth/Nose (%)	2 (1.77%)	2 (1.77%)	0	0
Fever (%)	1 (0.88%)	1 (0.88%)	0	0
Decreased Respiratory Function in Lungs (%)	1 (0.88%)	1 (0.88%)	0	0
Anal fissure (%)	1 (0.88%)	0	1 (0.88%)	0
Paronychia (%)	1 (0.88%)	1 (0.88%)	0	0
Gastritis (%)	1 (0.88%)	1 (0.88%)	0	0
Hyperuricemia/Cholesterol high (%)	1 (0.88%)	1 (0.88%)	0	0
Anemia (%)	2 (1.77%)	1 (0.88%)	0	1 (0.88%)

### Subgroup analysis

**FIGURE 3:**
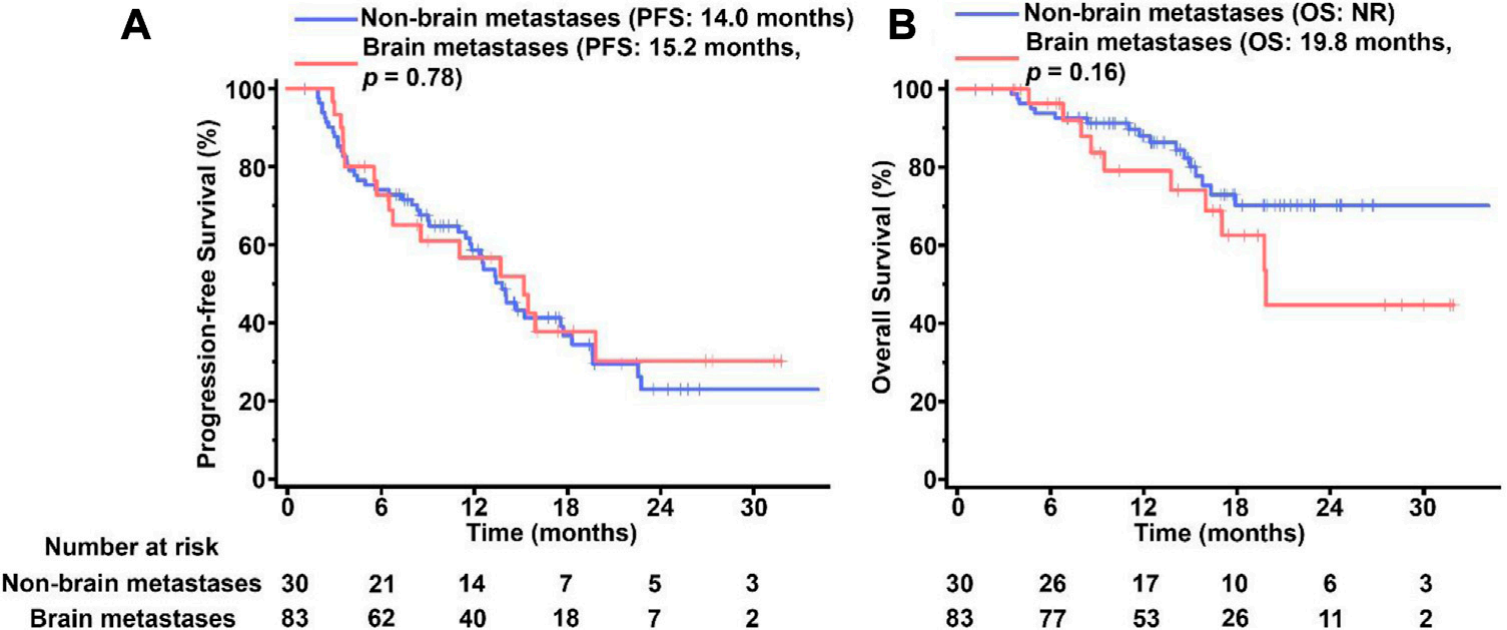
Kaplan-Meier survival of curves PFS **(A)** and OS **(B)** in pyrotinib-treated MBC patients with and without BM. The tick marks indicate the time points at which the data were censored. NR, not reached.

Brain metastases was found in 30 of the 113 pyrotinib-treated patients. In the patients with brain metastases, the median PFS was 15.2 months, which showed no significant difference from the median PFS in the patients without brain metastasis at 14.1 months (*p* = 0.76, Figure 3A). The overall OS in the patients without brain metastasis (not reachable) was better than that in the patients with brain metastasis (19.9 months) but did not reach a statistically significant level (*p* > 0.05, Figure 3B).

**FIGURE 4:**
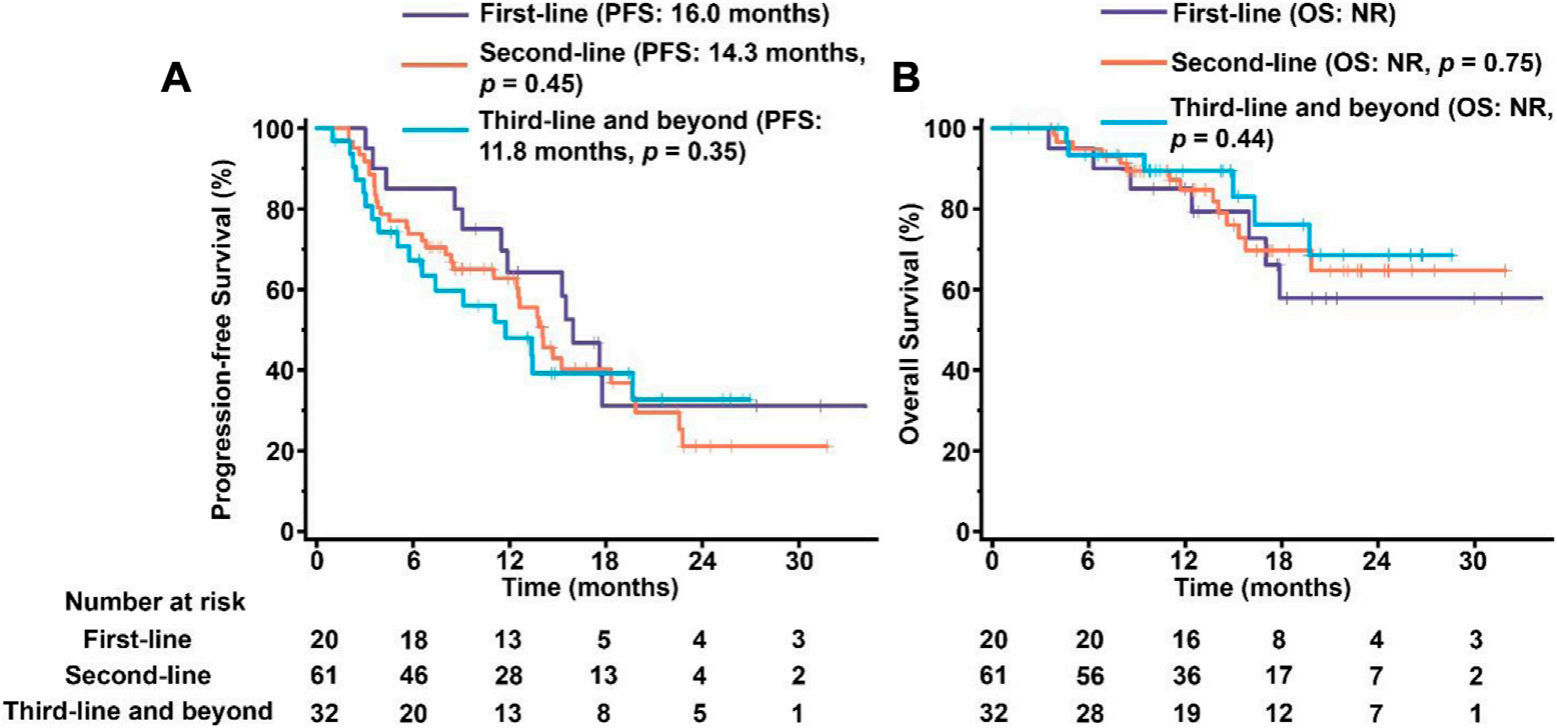
PFS **(A)** and OS **(B)** in patients treated by pyrotinib as first-line, second-line, third-line and beyond medicine. The tick marks indicate the time points at which the data were censored. NR, not reached.

We have also constructed Kaplan–Meier survival curves for different groups using pyrotinib as first-line, second-line, third-line or higher therapy. The majority of the enrolled patients (61 of 113) were treated by pyrotinib as the second-line medicine. Similar outcomes without significant differences were observed in three groups (*p* > 0.05, Figure 4), indicating that pyrotinib has the similar efficacy regardless of the stage of treatment.

## Discussion

In this prospective trial of 113 patients with HER-2 positive MBC, the median PFS was 14.1 months with an ORR of 66.4%. AEs were reported in most patients, but no grade 4 or higher TRAEs were observed. No significant differences in PFS and OS were found in subgroups with brain metastases or subgroups defined by the line of treatment. Our studies suggested that pyrotinib was effective in HER2+ MBC patients with brain metastases and patients from any line therapy could benefit from pyrotinib therapy.

For many years, HER2-positive breast cancer had worse prognoses and higher mortality rates compared to other subtypes of breast cancer (Owens et al., 2004; Cronin et al., 2010). Currently, the widespread use of anti-HER2 medications such as trastuzumab, pertuzumab, TDM1 and lapatinib greatly extended the median survival time of HER2-positive MBC patients (Eiger et al., 2021; Martinez-Saez and Prat, 2021). In this present study, our trial demonstrated encouraging benefits of pyrotinib therapy with a median PFS of 14.1 months and an ORR of 66.4%. Compared with the median PFS of 18.1 and 11.1 months and the ORR of 78.5% and 68.6% achieved by combination of pyrotinib plus capecitabine in prior phase II and III trials, our results are less encouraging. We suggested that the combination of pyrotinib with capecitabine was likely the main cause of the extended PFS and increased ORR. Therefore, it would be beneficial to explore the combination of pyrotinib with additional anti-HER2 therapies in future studies.

Several subgroup studies revealed that pyrotinib displayed better outcomes with patients receiving first-line treatment and pyrotinib should be applied as early as possible for patients with advanced HER2-positive. However, we did not observed any statistical significance in the PFS and OS of patients that were treated with pyrotinib plus capecitabine in different treatment lines, which suggested that pyrotinib plus capecitabine could be beneficial for HER2+ BC patients from any line therapy. Our results were also supported by previous real-world findings that the efficiency of pyrotinib was not related with number of pyrotinib lines. Therefore, we consider that the best treatment lines to receive pyrotinib for HER2-positive MBC still needs further inverstigation, especially in a large group study in multiple institutes.

Regarding safety, the incidence of grade 3 or 4 AEs resulting from chemotherapy was 30%–60% according to previous reports (Scagliotti et al., 2008; Kreuter et al., 2013), whereas pyrotinib led to grade 3 AEs in approximately 15% of individuals without causing any grade 4 or higher AEs in this study. The most frequent adverse events (AE) of tyrosine kinase inhibitors that target HER2/epidermal growth factor receptor is diarrhea (Chan, 2016; Ma et al., 2017). In this study, pyrotinib treatment cause diarrhea in 87.6% of patients, mainly grades 1 or 2, with 11.5% of patients developing grade-3 diarrhea. Diarrhea could be reversed with antidiarrheal medication, treatment interruption, or dose reduction, and it did not result in the termination of our study and previous treatments (Ma et al., 2017; Ma et al., 2019).

Although anti-HER2 monoclonal antibodies and HER2-directed antibody drug conjugates have been shown in several studies to prolong survival in patients with brain metastasis, their effects on the intracranial environment are still debatable because of the large-molecule property that prevents BBB infiltration (Garcia-Alvarez et al., 2021; Ferraro et al., 2022). One of our results with respect to HER2-positive MBC patients with brain metastases was that patients treated with pyrotinib had similar outcomes with patients without brain metastases, with an PFS of 15.2 months (Figure 2). According to a recent case study on patients with HER2-positive advanced gastric cancer and brain metastases, the combined treatment of pyrotinib, trastuzumab, and chemotherapy produced a PFS of 20 months, which is significantly longer than the median survival time of 2.9–6.2 months for most patients with advanced gastric cancer (Wang et al., 2022). Although more studies are required to comprehensively evaluate the efficacy and safety of pyrotinib in the HER2-positive MBC patients with brain metastases, our data suggested pyrotinib could also potentially benefit HER2-positive patients progressing on brain metastases.

There are several limitations in this study. First, this study’s sample size was relatively small and single-centered. The study design was not as rigorous as prior randomized trials. Second, the comparison between pyrotinib and other treatments was not possible because there was no control group. Finally, we did not further examine pyrotinib’s effects among patients with various levels of HER2 amplification because the majority of the patients in this research had high levels of HER2 amplification.

## Data Availability

The code and datasets analyzed during the present study are available from the corresponding authors upon request.
